# Sputum Smear Microscopy at Two Months into Continuation-Phase: Should It Be Done in All Patients with Sputum Smear-Positive Tuberculosis?

**DOI:** 10.1371/journal.pone.0039296

**Published:** 2012-06-19

**Authors:** Mohit Padamchand Gandhi, Ajay M. V. Kumar, Manoj Nandkishor Toshniwal, Raveendra H. R. Reddy, John E. Oeltmann, Sreenivas Achuthan Nair, Srinath Satyanarayana, Puneet Kumar Dewan, Shamim Mannan

**Affiliations:** 1 Office of the World Health Organization Representative in India, New Delhi, India; 2 South East Asia Regional Office, International Union Against Tuberculosis and Lung Disease, New Delhi, India; 3 Program for Appropriate Technology in Health, Hyderabad, India; 4 Division of Tuberculosis Elimination, National Center for HIV/AIDS, Viral Hepatitis, STD, and Tuberculosis Prevention, Centers for Disease Control and Prevention, Atlanta, Georgia, United States of America; 5 Central TB Division, Directorate General of Health services, Ministry of Health and Family Welfare, New Delhi, India; McGill University, Canada

## Abstract

**Background:**

The Revised National Tuberculosis Control Program (RNTCP) of India recommends follow-up sputum smear examination at two months into the continuation phase of treatment. The main intent of this (mid-CP) follow-up is to detect patients not responding to treatment around two-three months earlier than at the end of the treatment. However, the utility of mid-CP follow-up under programmatic conditions has been questioned. We undertook a multi-district study to determine if mid-CP follow-up is able to detect cases of treatment failures early among all types of patients with sputum smear-positive TB.

**Methodology:**

We reviewed existing records of patients with sputum smear-positive TB registered under the RNTCP in 43 districts across three states of India during a three month period in 2009. We estimated proportions of patients that could be detected as a case of treatment failure early, and assessed the impact of various policy options on laboratory workload and number needed to test to detect one case of treatment failure early.

**Results:**

Of 10055 cases, mid-CP follow-up was done in 6944 (69%) cases. Mid-CP follow-up could benefit 117/8015 (1.5%) new and 206/2040 (10%) previously-treated sputum smear-positive cases by detecting their treatment failure early. Under the current policy, 31 patients had to be tested to detect one case of treatment failure early. All cases of treatment failure would still be detected early if mid-CP follow-up were discontinued for new sputum smear-positive cases who become sputum smear-negative after the intensive-phase of treatment. This would reduce the related laboratory workload by 69% and only 10 patients would need to be tested to detect one case of treatment failure early.

**Conclusion:**

Discontinuation of mid-CP follow-up among new sputum smear-positive cases who become sputum smear-negative after completing the intensive-phase of treatment will reduce the laboratory workload without impacting overall early detection of cases of treatment failure.

## Introduction

The World Health Organization (WHO) and India’s Revised National Tuberculosis Control Programme (RNTCP) recommend periodic sputum smear microscopy during the course of tuberculosis (TB) treatment to monitor individual patient progress and assess overall programme performance [1], [2]. One of these follow-up microscopy examinations is scheduled between the 4^th^ and 6^th^ month of anti-TB treatment, i.e. in the middle of the ‘continuation phase’ (CP) of typical anti-TB treatment. The result of this (mid-CP) follow-up examination may have clinical implications; a patient found sputum smear-positive at the 5^th^ month of treatment (or later) is considered as not responding to treatment (‘treatment failure’) and is evaluated for possible drug resistance [2]. Further modification in treatment is based on results of drug susceptibility testing. Absence of mid-CP follow-up may therefore, in theory, result in a missed opportunity for evaluation for drug-resistance, a delay in treatment adjustments, and therefore poor treatment outcomes.

However, the utility of mid-CP follow-up in India has been questioned in a study from South India [3] where investigators concluded that mid-CP follow-up was not likely to be useful except in a small proportion of cases that remain sputum smear-positive at the end of the intensive phase of treatment (IP). This study could not influence policy on mid-CP follow-up, possibly because it was done in a small geographic area during the initial years of the RNTCP.

Discontinuation of mid-CP sputum smear examination offers the potential advantage of saving laboratory workload and patient inconvenience. The mid-CP follow-up accounts for about one-third of all the follow-up microscopy workload on laboratories. In India, patients must themselves bear the burden of lost time and travel expenses necessitated by each follow-up microscopy examination. With more than 800,000 sputum smear-positive cases registered under the RNTCP each year [4], the cumulative burden of this follow-up is substantial. We undertook a multi-district study to determine if mid-CP follow-up is of value to the RNTCP as measured by its contribution towards early detection of patients who are not responding to treatment.

## Methods

### Study Design

We conducted a descriptive study involving review of existing records maintained under the RNTCP.

### Setting

Bihar, a state in eastern India (population: 96 million), Maharashtra, a state in western India (population: 111 million), and Karnataka, a state in southern India (population: 59 million) together account for approximately one-sixth of sputum smear- positive cases notified in the country [4]. This study was conducted in five districts of Bihar, seven districts of Maharashtra and all 31 districts of Karnataka.

**Figure 1:**
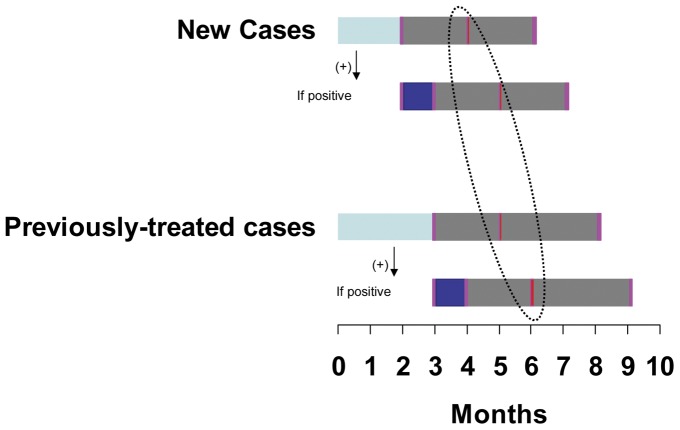
Timing of follow-up sputum smear examinations. Treatment duration and timing of follow-up sputum smear examinations for new and previously-treated cases of tuberculosis, highlighting the timing of the follow-up mid-continuation phase sputum smear examination. The initial two-three month intensive phase (light blue bars) is followed by a follow-up sputum smear examination (pink bars). If the follow-up sputum smear examination is positive, an additional 1-month extended-intensive phase treatment is given (dark blue bar), with additional follow-up sputum smear examination. After the intensive phase (or extended intensive phase), the continuation phase of treatment (grey bars) is immediately begin. After two months of continuation phase (i.e. between months 4 and 6), the mid-continuation phase (mid-CP) follow-up sputum smear examination is done (red bars, circled with dotted line). At the end of the continuation phase, a final follow-up sputum smear examination is done.

Under the RNTCP, all patients with TB are treated with a standardized short-course thrice-weekly intermittent regimen under direct observation of a trained (DOT) provider. The treatment consists of an intensive phase (IP) consisting of four to five drugs in the initial two to three months and a continuation phase (CP) consisting of two to three drugs for four to five months, depending on the history of previous treatment. In addition, cases that are sputum smear-positive at the end of IP, receive a four week extension of the IP regimen. Follow-up sputum smear microscopy is recommended at the end of IP, extended-IP, mid-CP and at the end of treatment to assess patients’ clinical progress (Figure 1). The DOT provider reminds and motivates the patient to undergo the prescribed sputum smear follow-ups at a Designated Microscopy Center (DMC) and updates a treatment card with the follow-up results. The outcome is assessed by a Medical Officer on the basis of record of treatment and follow-ups maintained in the treatment cards. All these events, from initiation of treatment to various follow-ups and finally, the outcome, are transcribed into a TB register by the treatment supervisor of that reporting unit. The quality of sputum smear microscopy is assured by laboratory supervisors at the district through a monthly random blinded quality assurance process.

### Study Period

We conducted the study during September 2010 to August 2011.

**Figure 2:**
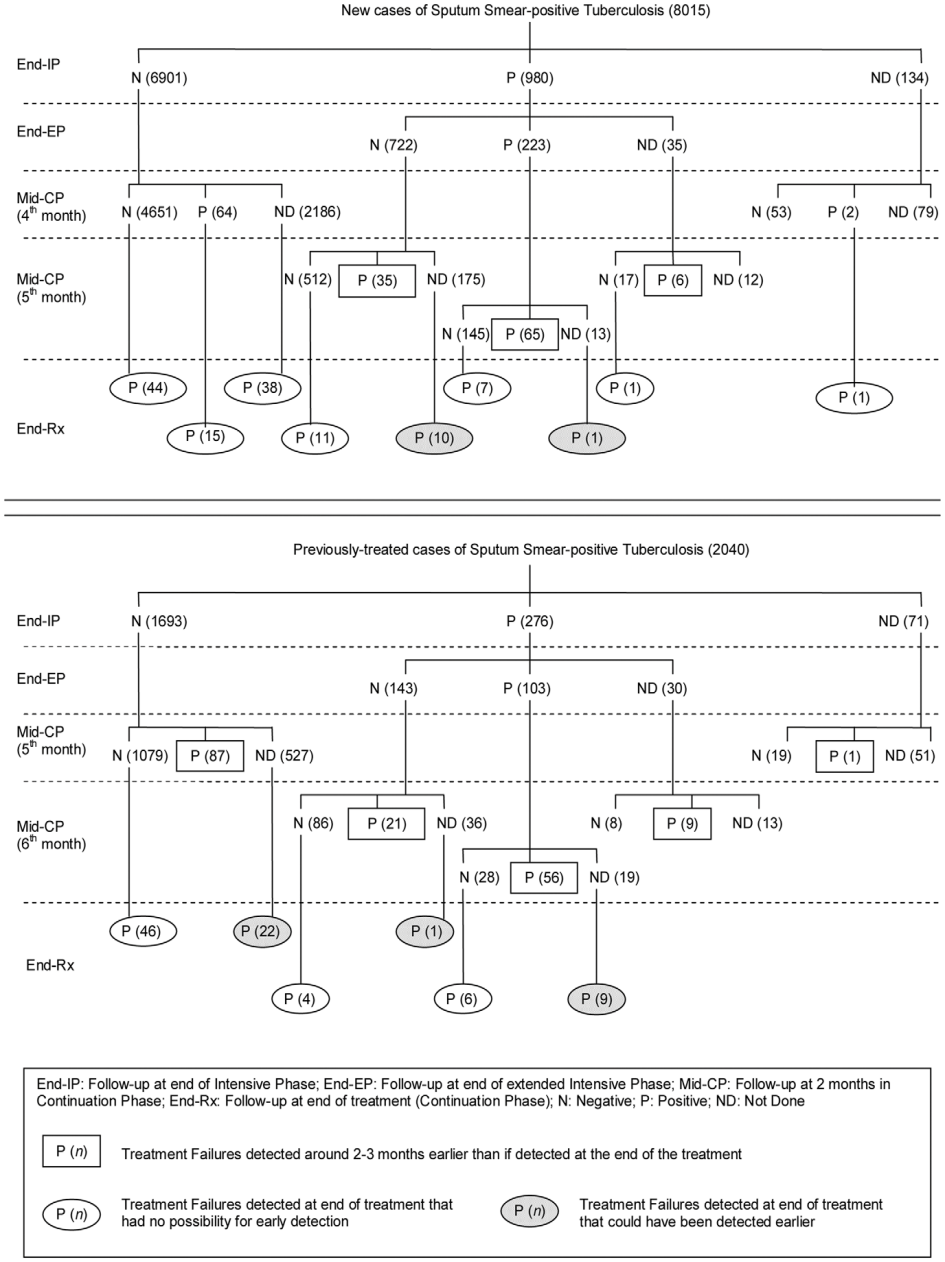
Flow of cases of sputum smear-positive Tuberculosis as per successive sputum smear microscopy results. Mid-CP follow-up refers to the sputum smear microscopy which is scheduled between the 4^th^ and 6^th^ month of anti-TB treatment, i.e. in the middle of the ‘continuation phase’ (CP) of typical anti-TB treatment. Cases that are found to be smear-positive at 5^th^ month or later are considered as not responding to treatment (‘treatment failure’). Numerals shown in rectangular boxes refer to number of cases with treatment failure who were detected two-three months earlier than at the end of treatment as they were smear-positive in mid-CP follow-up at or beyond 5^th^ month of anti-TB treatment. Numerals shown in clear ovals refer to number of cases with treatment failure that were detected only at the end of treatment because they were either smear-negative in mid CP follow-up, or had a smear-positive mid-CP result before 5^th^ month of anti-TB treatment. Numerals shown in shaded ovals refer to number of cases with treatment failure who missed their mid-CP follow-up scheduled at or beyond 5^th^ month of treatment. These cases could have been detected two-three months earlier had they undergone mid-CP follow-up and were found to be smear-positive.

### Study Population

All cases of sputum smear-positive TB registered for treatment under the RNTCP during a three month period in 2009 [July to September (Maharashtra and Bihar); April to June (Karnataka)] in all the reporting units of selected districts were included in the study. However, cases that were registered for treatment elsewhere and later, got transferred-in [4] to the study reporting units, and those that were prescribed Non-DOTS treatment regimen [4], were excluded.

### Sampling

No sampling was done. All eligible cases of sputum smear-positive TB registered during the study period were included in the study. Selection of States, districts and study period were based on feasibility of data collection.

### Study Variables

Data on type of case (new or previously-treated), result of sputum smear microscopy at the end of IP, at the end of extended IP (wherever applicable), at two months into CP and at end of treatment, and outcome of treatment were collected. Results of sputum smear microscopy were recorded as negative, positive, not applicable, or not done. A positive result included all registered grades (3+, 2+, 1+ or Scanty) as defined under the program [4]. Missing entries with respect to follow-up results were recorded as not applicable (if a patient could not be expected to undergo a particular follow-up on account of death, default, failure or transfer to another reporting unit), or, as not done. Outcome of treatment was recorded as cured, treatment completed, died, default, failure and transfer-out, as per the standard WHO definitions [1]. If a case of treatment failure was detected based on sputum smear-positivity at mid-CP follow-up rather than at the end of treatment, it was considered as detected early. Conceptually, ‘early’ refers to the time gap between the mid-CP and the end of treatment sputum smear examination, and equates to around two months for new cases, and three months for previously-treated cases (Figure 1).

### Data Collection and Entry

In Bihar, the required data were extracted into a pre-structured data collection format by the principal investigator from TB registers after validating the results of follow-up sputum smear examinations with TB laboratory registers. Data were then doubly entered in Epi-data (Version 3.1) and the two data-sets were compared for discrepancies, which were resolved by referring to the hard copies of original data collection formats. The final database was exported into MS Excel. Validated data from Karnataka and Maharashtra were received in a similar format. The three data-sets were finally collated and analyzed.

**Table 1:** Impact of various policy options with regard to mid-CP follow-up on the number of cases of tuberculosis treatment failures detected early^1^ and laboratory workload.

Policy Options for mid-CP follow-up	Total patients tested (%)^2^	Cases of TreatmentFailure detected early^1^(Gain/Loss)^3^	Number needed to testto detect one case oftreatment failure early^1^
Continue as per present guideline	10055 (100)	323	31
Discontinue in new end-IP negative cases	3154 (31)	323	10
Discontinue in all new cases	2040 (20)	206 (−117)	10
Discontinue in all end-IP negative cases	1531 (15)	214 (−109)	7
Schedule mid-CP follow-up to 5th month for all cases	10055 (100)	421 (+98)	24

1 Detected as a case of treatment failure around two-three months earlier than if detected at the end of the treatment.

2 Percentage calculated by taking follow-up required under present policy as denominator.

3 Gain/Loss in absolute numbers indicated respectively by a positive or a negative sign; CP-Continuation Phase of TB treatment.

### Data Analysis

We calculated the proportion of cases that underwent mid-CP follow-up and the proportion of cases that were detected early as treatment failure based on mid-CP follow-up. We also estimated the additional number of cases detected early as treatment failure if all cases had undergone mid-CP follow-up, assuming that all cases with positive sputum smears at the end of treatment would also have been sputum smear-positive in mid-CP follow-up. Lastly, we estimated the number needed to test to detect one case of treatment failure early, with different policy options related to mid-CP follow-up.

### Ethics Approval

Under the RNCTP, written consent of patient for storage and future usage of his/her personal details is not taken as a routine. However, personal details of patients are not shared with anybody outside the care, support and treatment framework, without prior consent of the patient.

For this study, which involved a review of routinely maintained records under the RNTCP and did not involve patient interaction in the process of data collection, individual patient consent was not necessary. The study protocol was examined and approved, and individual patient consent was determined to be unnecessary by the Ethics Advisory Group of International Union Against Tuberculosis and Lung Disease, and the Ethics Committee of the National Tuberculosis Institute, Bangalore, India. Participation of U.S. Centers for Disease Control and Prevention (CDC) in this project did not meet the definition of engagement in human subjects’ research and separate institutional review board approval was not required.

## Results

A total of 12,558 cases of sputum smear-positive TB were included in the study. As a result of death, default or transfer to some other reporting unit, 2503 (20%) cases did not reach the 2^nd^ month of CP (i.e. the time when mid-CP follow-up becomes due under the RNTCP). Of the remaining 10,055 cases, all of which should have gotten mid-CP follow-up examination as per the RNTCP guidelines, mid-CP follow-up was documented in 6944 (69%) cases. Of these, 280 (4%) cases were detected as treatment failure on the basis of mid-CP follow-up, around two-three months earlier than if detected on the basis of sputum smear examination at the end of the treatment.

Of the 8015 new cases with sputum smear-positive TB, 5550 (69%) cases underwent mid-CP follow-up. Of these, 106 (2%) were detected early as cases of treatment failure (Figure 2). If the remaining 2465 cases had undergone mid-CP follow-up, and assuming that all cases with positive sputum smears at the end of treatment would also have been sputum smear-positive in mid-CP follow-up, an estimated maximum of 11 additional cases of treatment failure could have been detected early (Figure 2). So, at best, mid-CP follow-up could have benefitted 117 of 8015 (1.5%) new cases.

Of the 2040 previously-treated cases with sputum smear-positive TB, 1394 (68%) cases underwent mid-CP follow-up. Of these, 174 (12%) were detected early as cases of treatment failure (Figure 2). If the remaining 646 cases had undergone mid-CP follow-up, and assuming that all cases with positive sputum smears at the end of treatment would also have been sputum smear-positive in mid-CP follow-up, an estimated maximum of 32 additional cases of treatment failure could have been detected early (Figure 2). So, at best, mid-CP follow-up could have benefitted up to 206 of 2040 (10%) previously-treated cases.

Overall, if all 10,055 cases with sputum smear-positive TB had undergone mid-CP follow-up, up to 323 (3.2%) cases could have benefitted from mid-CP follow-up.

As per present policy, about 31 patients need to be tested by mid-CP follow-up to detect one case of treatment failure early (Table 1). Discontinuation of mid-CP follow-up in new sputum smear-positive cases who were sputum smear-negative at the end of IP would achieve the same at a much reduced effort with a number needed to test of just 10. However, discontinuation of mid-CP follow-up in all cases (new and previously-treated) who became sputum smear-negative at end of IP (end-IP negative), or, in all new cases, could have led to a delay in declaration of 109 to 117 cases, respectively, as treatment failure (Table 1; Figure 2). By scheduling mid-CP follow-up at the 5^th^ month, 98 additional cases of treatment failure could have been detected early without increasing the overall laboratory workload (Table 1; Figure 2).

Discontinuation of mid-CP follow-up among the 6901 new sputum smear-positive cases who were sputum smear-negative at the end of IP would delay by two months the opportunity of evaluation of possible drug resistance for 64 (<1%) cases that were found sputum smear-positive in mid-CP follow-up.

## Discussion

We found that, at best, only 117/8015 (1.5%) new and 206/2040 (10%) previously-treated cases of sputum smear-positive TB could have benefitted from follow-up sputum smear examination at two months into the continuation phase of treatment (mid-CP follow-up). As per present policy, the program needs to undertake mid-CP follow-up in about 31 cases to detect one case of treatment failure early. The number needed to test to detect a single case of treatment failure early could be reduced to as low as 7 (up to 77% reduction) by considering different policy options for mid-CP follow-up. Given the burden of this follow-up under the RNTCP, the program should consider a policy change.

Discontinuing mid-CP follow-up among new sputum smear-positive TB cases whose sputum smear is negative at the end of IP appears to be the best option, because a similar number of cases of treatment failure would have been detected early as detected under current policy while reducing the total workload by two-thirds. This policy option would also have substantially reduced the patient-incurred costs involved in travelling to a microscopy centre for follow-up sputum smear examination.

Discontinuation of mid-CP follow-up among all new cases, or among all (new and previously-treated) end-IP sputum smear-negative cases, would have decreased the overall laboratory workload as well as the effort required to detect one case of treatment failure early. However, this could have led to two-three months delay in detection of a significant number of cases of treatment failure, thus, delaying drug susceptibility testing and necessary treatment adjustments.

Scheduling mid-CP follow-up sputum smear examination at the 5^th^ month instead of two months into CP is a tempting option. This policy change would have allowed for early detection of 98 additional cases of treatment failure without increasing the laboratory workload any further. But ascertaining the 5^th^ month of treatment may be difficult for the DOT provider. The current recommended practice is to send the patient for mid-CP follow-up at the time of dispensing the 8^th^ weekly blister-pack, which roughly equals to two months into CP. Counting empty blister-packs is easier than counting months from the date of starting treatment for the DOT providers who may have limited education.

As the present guideline of the RNTCP is to evaluate each follow-up sputum-smear positive case for possible drug resistance, will the recommended policy change lead to missed opportunities for detecting multi-drug resistance? The study data suggest that this could have happened for a small minority [∼64/6901 (1%)], but these cases would have had another opportunity two months later, if found sputum smear-positive at the end of treatment. We believe this risk to be worth taking, given the substantial reduction in laboratory workload.

This study was conducted in 170 reporting units of 43 districts spread across three states of the country and we believe that it is indicative of the national situation. A key limitation of this study is that uptake of mid-CP follow-up was low (69%), as is the reality under routine programme conditions. For this reason, we estimated the additional benefit by assuming the best case scenario, and still found little utility of mid-CP follow-up among new cases with sputum smear-positive TB. The reasons for the low uptake of mid-CP follow-up were beyond the scope of the current study and need to be addressed by future research.

### Conclusion

We believe the national TB control programme in India should consider a change in policy regarding mid-CP follow-up. Taking program priorities and field conditions into view, discontinuation of mid-CP follow-up among those new sputum smear-positive TB cases who become sputum smear-negative after the intensive-phase of treatment, appears to be the best option. This will save human and material resources for the program, without delaying identification of multidrug resistant cases.
